# Ionic Liquid@Metal-Organic Framework as a Solid Electrolyte in a Lithium-Ion Battery: Current Performance and Perspective at Molecular Level

**DOI:** 10.3390/nano12071076

**Published:** 2022-03-25

**Authors:** Mohd Faridzuan Majid, Hayyiratul Fatimah Mohd Zaid, Chong Fai Kait, Azizan Ahmad, Khairulazhar Jumbri

**Affiliations:** 1 Department of Fundamental and Applied Sciences, PETRONAS Technology University, Seri Iskandar 32610, Perak Darul Ridzuan, Malaysia; mohd._17006281@utp.edu.my (M.F.M.); chongfaikait@utp.edu.my (C.F.K.); khairulazhar.jumbri@utp.edu.my (K.J.); 2 Chemical Engineering Department, PETRONAS Technology University, Seri Iskandar 32610, Perak Darul Ridzuan, Malaysia; 3 Centre of Innovative Nanostructures and Nanodevices (COINN), Institute of Autonomous System (IAS), PETRONAS Technology University, Seri Iskandar 32610, Perak Darul Ridzuan, Malaysia; 4 Department of Chemical Sciences, The National University of Malaysia, Bangi 43600, Selangor, Malaysia; azizan@ukm.edu.my; 5 Department of Physics, Faculty of Science and Technology, Airlangga University (Campus C), Mulyorejo Road, Surabaya 60115, Indonesia; 6 Centre for Research in Ionic Liquids (CORIL), Institute of Contaminant Management (ICM), PETRONAS Technology University, Seri Iskandar 32610, Perak Darul Ridzuan, Malaysia

**Keywords:** ionic liquid, metal-organic framework, solid-state electrolyte, lithium-ion battery, ionic conductivity

## Abstract

Searching for a suitable electrolyte in a lithium-ion battery is a challenging task. The electrolyte must not only be chemically and mechanically stable, but also be able to transport lithium ions efficiently. Ionic liquid incorporated into a metal–organic framework (IL@MOF) has currently emerged as an interesting class of hybrid material that could offer excellent electrochemical properties. However, the understanding of the mechanism and factors that govern its fast ionic conduction is crucial as well. In this review, the characteristics and potential use of IL@MOF as an electrolyte in a lithium-ion battery are highlighted. The importance of computational methods is emphasized as a comprehensive tool to investigate the atomistic behavior of IL@MOF and its interaction in electrochemical environments.

## 1. Introduction

In 2020, a report entitled “*Innovation in batteries and electricity storage—A global analysis based on patent data*” states that a battery contributes to nearly 90% of all patenting activity in electrical storage. The increase in this advancement is mainly driven by the development of rechargeable lithium-ion batteries (LIBs) utilized in consumer portable electric devices and electrical cars. LIBs have recently attracted great attention owing to their high energy density compared to other available rechargeable battery technologies, such as lead-acid battery and nickel–cadmium battery. LIBs were introduced to the market in 1991 by Sony (Tokyo, Japan), Toshiba (Tokyo, Japan) and Asashi Kasei C (Tokyo, Japan). The battery used lithium cobalt oxide, a kind of layered oxide chemistry specifically for the design of cathode material. In 2019, John B. Goodenough, M. Stanley Whittingham and Akira Yoshino were awarded the Nobel Prize in Chemistry thanks to their pioneering work in LIBs. According to Web of Science data analysis, the popularity of LIBs in academia is shown by the rising number of research articles with an increase of 10.523% in the last 10 years. Battery experts believe that the attractiveness of LIBs lies on its electrodes, which are not strongly influenced by chemical reaction that may deteriorate the overall performance of the battery. Another unique criterion is the impressive energy density of LIBs (100 to 265 Wh/kg) compared to other commercial rechargeable batteries. Moreover, LIBs only require short charging times and can be used for many cycles without sacrificing its lifespan. LIBs are commonly used as a power supply in portable electronic devices, such as smartphones, laptops and tablets [1,2,3]. Most recently, LIBs have grown in popularity in electrical vehicles [4,5,6], military devices [7,8] and aerospace industry [9,10,11]. In one report, the demand for LIBs in the electrical vehicle industry exponentially increased from 0.5 GWh to 526 GWh in the last decade. It is also expected that the supply for LIB will grow by 17-fold by 2030, which will bring the cost of battery storage down [12]. In fact, Japan and Korea are currently the major players of the global battery technology, whereby the recent advancement and bulk production in the battery industry led to a remarkable drop in battery prices of up to 90% since 2010 for the electric vehicle sector. The current state of the art of LIBs is focusing on the development and advancement of new material, particularly for the design of electrodes and electrolytes. Currently, the reliable material for cathodes utilizes lithium transition metal phosphates, LiFePO_4_, and lithium transition metal oxides, LiCoO_2_. The commercially dominating anode materials are graphite and lithium titanate [13]. Battery experts also anticipate the transition to alloyed silicon and silicon oxide, SiO_x_, because of their higher specific capacities compared to carbon-based anodes [14,15,16,17,18]. Although the development of electrode materials for LIBs is actively progressing [19,20,21,22,23], research in electrolyte has also gained interest from both academic and industry practitioners [24,25,26,27]. One of the main components of LIBs that plays an important role is electrolytes. An electrolyte will facilitate the movement of lithium ions from anode to cathode (discharging) and vice versa (charging) through a separator. When ions flow, free electrons are formed in the anode which creates charge at the positive current collector. This generates an electrical current flowing from the current collector through a plugged device and the negative current collector. The electrolyte components for commercial purposes consist of lithium hexafluorophosphate, LiPF_6_, as conducting salt, a mixture of carbonates (dimethyl carbonate, DMC, ethyl methyl carbonate, EMC, diethyl carbonate, DEC, and ethylene carbonate, EC) as solvents and fluoroethylene carbonate, FEC, or vinylene carbonate, VC, as additives. The choice of this composition is based on several factors: (i) the low dissociation energy of LiPF_6_, which is highly stable in carbonate solution; (ii) the high stability of hexafluorophosphate ions towards oxidation, which allow the utilization of a high potential cathode (more than 4 V); (iii) the low susceptibility towards corrosion at a high potential; and (iv) the presence of a solid electrolyte interphase (SEI) from the electrochemical reduction of additives and solvents, which allow the intercalation and deintercalation of lithium cations. However, the LiPF_6_ electrolyte system suffers from thermal decomposition at low temperatures due to the trace amount of moisture that induces the degradation of the electrolyte. Furthermore, the use of a flammable volatile organic solvent has raised safety concerns among consumers. Because of the strong reductant and oxidant properties of the anode and cathode, by utilizing a liquid-based electrolyte, the risk of thermal runaway is unavoidable, which can cause the emission of toxic gases and explosions that may result in unwanted incidents [28].

Efforts have been made to address the flammability concerns regarding the usage of a liquid electrolyte by discovering other safe alternatives to anions, such as phosphates, borates, imides and Hückel-type salts [29,30,31,32,33,34,35,36,37]. Incorporating hydrophilic lithium bis(trifluoromethanesulfonyl)imide, LiTFSI, into the electrolyte system is another strategy as it acts as a co-salt [38,39]. Nonetheless, the replacement of the solution electrolyte to a solid-state electrolyte is more promising as it allows greater safety, improved thermal stability, non-volatility and affordable power density and cyclability [40,41,42,43,44,45,46,47]. It is also non-flammable, which could prevent electrolyte leakage, short circuit and can circumvent ohmic polarization in LIBs.

The state of the art of SSE is mainly focused on the development of inorganic solid electrolytes (ISEs), solid polymer electrolytes (SPEs), composite polymer electrolytes (CPEs) and gel polymer electrolytes (GPEs). The first ISEs—silver sulfide, Ag_2_S, and lead(II) fluoride, PbF_2_—were studied by M. Faraday in the nineteenth century [48,49]. Inorganic ceramic is usually in crystalline or glassy form and its skeleton structure allows lithium ions to move and diffuse within their lattice structure. They have high ionic conductivity ranges from 10^−4^ S cm^−1^ up to 10^−2^ S cm^−1^, a high lithium transference number and high modulus; however, they are prone to mechanical fracture and become problematic when considering electrode–electrolyte compatibility. The current state-of-the-art ISEs are commonly prepared from phosphates, sulfides and oxides crystal structures, including lithium superionic conductors (LISICONs), garnets (LLZO), argyrodite, hydrides and perovskites. Meanwhile, SPEs are made of a solvent-free salt solution in a polymer matrix, which conducts ions via the interaction of functional groups within the polymer chain. Peter V. Wright was the first to discover a polyethylene oxide (PEO) that can conduct lithium ions through a polymeric network. Nowadays, PEO is widely used as polymer hosts in the development of SSE due to its superior energy density, ease in fabrication, safeness, economic value, high electrochemical stability and good compatibility with lithium salts [50]. Synthetic polymers, such as polyethers, polyesters, polycarbonates and fluoropolymers, are also used to form SPE. Moreover, research towards sustainable bio-resources, including cellulose, lignin and chitosan, have also gained popularity in SPE synthesis. Although the preparation of SPE is easy, has high elasticity and stable interface with electrodes, the weakness of SPE is its low ionic conductivity (less than 10^−4^ S cm^−1^), especially at low room temperature, and thus this type of SSE is usually operated at elevated temperatures, limiting its practical application in ambient temperature [51,52,53]. Even if complexed with lithium salt, the amorphous structure of PEO, which is the main driving force of the fast ionic transport of LIBs, is transformed into a crystalline phase below 60 °C. Modifying the structure of the polymer matrix to retain its glass-forming ability at low temperatures is necessary to improve ionic conductivity. This is achieved by introducing solid fillers, metal salts and plasticizers to form CPEs [54,55,56,57,58]. The main advantages of CPEs are its improved interfacial contact, better flexibility and economics. One of the main concerns when using CPEs is their low mechanical stability because of poor elasticity. The insertion of inorganic ceramic fillers that are inert to lithium conduction within a polymer matrix could improve the mechanical structure of CPEs. The presence of fillers, such as titanium dioxide (TiO_2_), silicon dioxide (SiO_2_) and aluminum oxide (Al_2_O_3_), destructs polymer crystallinity, which eventually could enhance the ionic conductivity of the electrolyte [59]. On the other hand, GPEs are obtained from the addition of low molecular weight plasticizers or organic electrolytes to a polymer network [60]. The electrolyte system has two distinctive regions—the ionically conductive gel polymer and the rigid polymer matrix that retains the electrolyte structure [60,61,62,63]. Therefore, GPEs have both characteristics of solid and liquid electrolytes and thus possess good mechanical stability and high ionic conductivity. Nonetheless, the release of volatile compounds in GPEs increases the electrolyte–electrode reactivity [64], therefore, the careful selection of plasticizer and organic compounds is crucial to allow for the usefulness of GPEs.

New formulations of SSE are needed to surpass the limitations and performance of the available SSE systems. More recently, metal–organic frameworks (MOFs) have emerged as a promising candidate for the improvement of LIB manufacturing. MOFs are three-dimensional crystalline materials that are made from the coordination interaction of transition metal ions or clusters and multidentate organic ligands. The main properties of MOFs are their high porosity (up to 90%) and vast internal surface area (6000 m^2^/g) [65]. The pore structure can be modified by the suitable selection of precursors that offers high designability and functionality to MOFs. Their tunable porosity is suitable for many applications in storage and host–guest interactions, including energy storage, energy conversion, catalysis, separation and gas adsorptions. Compared to the commercially available electrolytes, MOFs have the benefits of ionic conduction enhancement, adjustable organic linkers and metal nodes, a hierarchical framework and scalable processing.

Owing to their unique porosity properties, MOFs could enable host–guest chemistry via the insertion of guest ions or molecules to improve electrochemical performance. The host–guest complex is formed via non-covalent bonding, which is critical in retaining the 3D structure of MOFs. Various protonic molecules have been tested, including imidazole, triazole, classes of ionic liquids (ILs), histamine and ammonium salt. Among these, ILs could offer more benefits due to their low flammability, low volatility, thermally stable and up to 6.0 V of electrochemical window, when compared to conventional organic electrolytes. Although ILs are in liquid form, when impregnated into MOFs, the mobility of ILs is no longer the same as in bulk ions. The concept of confining ILs into a nanoporous MOF (IL@MOF) has gained much attention especially in energy storage applications due to their solvent-free properties, which could mitigate liquid leakages [66]. Moreover, a recent study by Fujie et al. showed that there was no phase transition of IL@MOFs even at low temperatures when compared to pristine MOFs. The IL@MOF showed higher ionic conductivity compared to the bulk IL because of the nanosized ILs in MOF micropores, which prevents freezing transition. This could open a new possibility to design a flexible LIB device that can withstand extreme temperatures, while eliminating the risk of leakages [67].

The demand of high-energy density LIBs is rapidly increasing due to the fast development in portable electronics and electrical vehicles. Most of the commercially available LIBs utilize liquid electrolytes, which raise safety concerns towards consumers. Replacing liquid electrolyte with SSEs is one of the initiatives to manufacture safe and reliable future generation storage devices. Efforts have been made to fabricate an all-solid-state-battery using SSE that possesses low reactivity with electrodes that could sustain the lifetime of LIBs. The potential use of SSEs to conduct electricity at relatively low temperatures without diminishing the performance of lithium diffusion has also been demonstrated by utilizing nanoconfined IL@MOF.

Nonetheless, there are some characteristics of IL@MOFs that are poorly understood when designing stable LIBs. Firstly, the mechanical stability of IL@MOFs influences the interface formation between SSEs and cathodes. Since most of the studied SSEs have low deformation, this may result in mechanical particle-to-particle contact loss. This happens due to the expansion and contraction of the cathode over cycling, which leads to periodic structure and volume change. Secondly, the chemical reaction between the IL@MOF and the electrode is another factor that needs to be considered in the manufacturing of LIBs. A severe interface impedance might occur if the candidate SSE possesses high reactivity and low wetting performance towards the lithium metal anode. Thirdly, another concern when utilizing LIBs is the growth of dendrites, which can cause unwanted reactions in the electrolyte–electrode interface. Dendrite formation within LIBs should be minimized to realize saleable high-energy density SSEs.

## 2. Working Principles of Lithium-Ion Batteries

LIBs work differently compared to conventional electrochemical cells. Instead of relying on redox reactions, LIBs mainly operate via intercalation processes, which involve the reversible inclusion of lithium ions into a vacant site of the crystalline lattice structure of the electrode host. The electrode materials are usually in the form of an open crystal structure or layered structure, such as graphite, C_6_, in the anode or oxide from *d*-block elements, such as lithium cobalt oxide, LiCoO_2_, in the cathode. The process of intercalation accommodates lithium ions and electrons at the same time without altering the crystal structure of the electrode.

The sources of lithium are stored in electrodes and the diffusion of lithium is facilitated by electrolytes. As in electrochemical cells, the oxidation process occurs at the negative electrode (anode), while the reduction process occurs at the positive electrode (cathode). However, the “anode” and “cathode” terms are both interchangeable, depending on whether it is a charging or discharging process. During the charging process, the lithium is oxidized from LiCoO_2_ into lithium ions and electrons. Lithium ions are then diffused through the electrolyte, eventually intercalated and stored into the C_6_ multi-layer sandwich. As the battery discharges, lithium ions are deintercalated from the negative electrode, migrating through the electrolyte and inserted back into the lattice structure of the positive electrode. At the same time, electrons travel across the external circuit, generating electricity to the electrical supply, which is accepted by the positive electrode. Because the process is entirely reversible, the lithium ions shunt back and forth between electrodes. The reversible charging–discharging process can be described by half-cell reactions as shown below:

- Oxidation reaction: LiC_6_ (s) → C_6_ (s) + Li^+^ (aq) + e^−^
- Reduction reaction: CoO_2_ (s) + Li^+^ (aq) + e^−^ → LiCoO_2_ (s)
- Overall: LiC_6_ (s) + CoO_2_ (s) ⇌ C_6_ (s) + LiCoO_2_ (s).

Common LIBs utilize LiC_6_ as the negative electrode. The layered structure of the graphene sheet is designed in such a way that allows the storage and discharge of lithium ions via intercalation. The layered LiCoO_2_ is commonly used as the positive electrode. Additionally, polyanion material, such as lithium iron phosphate, LiFePO_4_, is also used as positive electrode due to its low cost and minimum toxicity. Other positive electrodes, such as spinel oxide (lithium manganese oxide, LiMn_2_O_4_), have also been utilized. The different lattice structure in these cathodes allows the diffusion of lithium ions in a distinct fashion. The electrolyte consists of a mixture of a salt, acid or base in a solvent. Lithium hexafluorophosphate, LiPF_6_, lithium tetrafluoroborate, LiBF_4_, and lithium perchlorate, LiClO_4_, are conventionally used as electrolyte salt. Solvents accompanying the liquid electrolytes are usually of the carbonate family, such as ethylene carbonate, EC, propylene carbonate, PC, dimethyl carbonate, DMC, ethyl methyl carbonate, EMC, and diethyl carbonate, DEC.

One of the advantages of LIBs is that it provides high energy density due to their high capacitance to power electrical devices. When compared to other rechargeable battery, such as lead acid battery, LIBs are efficient in power storage. They can be recharged for multiple times and still be able to perform fast charging. Moreover, LIBs have minimum self-discharge. In battery technology, self-discharge is a chemical reaction that occurs inside a battery, which brings down the stored charge. A low self-discharge improves the shelf life of storage devices. Additionally, the maintenance of LIBs is low, which could benefit their prolonged performance. The main reason for this is because LIBs do not exhibit memory effects, as it happens in nickel–cadmium batteries. Such a phenomenon results in a gradual capacity loss, which requires the careful maintenance to preserve the performance of a battery.

Nevertheless, since most commercial LIBs use a liquid material as an electrolyte, they have a propensity towards battery leakage. LIBs normally employ carbonates as solvent in the electrolyte mixture; however, they are flammable and susceptible to ignition, which could lead to unwanted fire incidents. For instance, several explosions of Samsung Galaxy Note 7 smartphones reported in 2016 and Tesla Model S blast incidents in 2019 are some of the cases involving faulty LIBs [68]. Furthermore, another issue of liquid-loaded electrolytes is the narrow range of working temperatures, which may hinder the applicability of this battery under extreme temperatures. For example, in high temperature conditions, the heat released induces electrolyte coagulation and vaporization. The gas production may react with the surrounding environment, producing a fuel–air mixture that is easy to explode [69]. All these issues have raised safety concerns among LIB manufacturers and end consumers and, therefore, switching conventional liquid-based LIBs into all-solid-state LIBs is crucial to ensure the high reliability of LIB technology.

**Figure 1:**
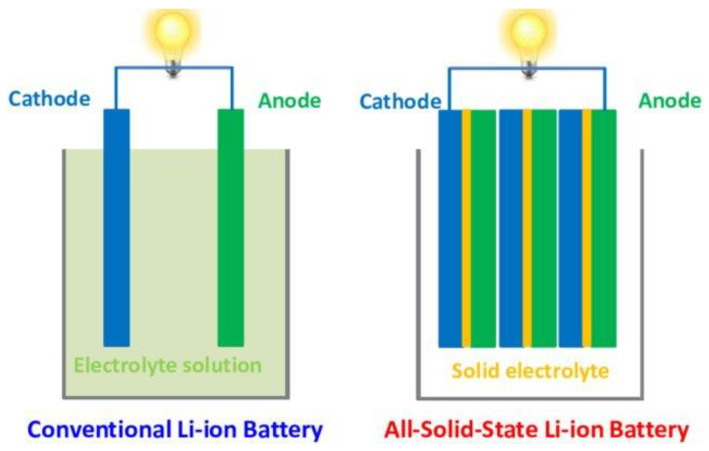
Schematic diagram of a conventional LIB (**left**) and an all-solid-state LIB (**right**). Reprinted from [76].

Figure 1 illustrates the different setup of conventional LIBs and solid-state LIBs. The all-solid-state LIBs are made of entirely solid materials for the electrode and electrolyte components. Since there is no liquid present, the likelihood of explosion and electrolyte volatility could be alleviated, offering safer and more stable LIBs. The manufacturing of LIBs can be simplified via thin-filming processing, which eventually increase their specific energy, energy density and power density. The existing materials for SSEs include ICEs, SPEs, CPEs and GPEs. Utilizing SSEs allow wide operating temperatures, longer cycle life compared to liquid electrolyte, high electrochemical stability and minimize dendrites growth. However, there are still existing problems when employing these SSEs in a LIB system. Some of the SSEs, such as SPE, still lack physical and chemical stability, which might be an issue in battery assembly [70,71]. Moreover, sluggish ionic diffusion in certain ICE might restrict charge–discharge rate capabilities [72,73]. Additionally, large volumetric changes may cause mechanical instability, which may disrupt the overall performance of LIBs [40]. Nanomaterials could offer an alternative strategy to redesign a stable and powerful ionic conductor. In this research, IL@MOF, a 3D porous frameworks confined with IL, is utilized as a SSE candidate due to its tunable porosity and large volumetric storage properties, which is suitable in manufacturing new SSEs [74,75].

## 3. Ionic Liquid@Metal–Organic Framework (IL@MOF) as a Solid-State Electrolyte

IL@MOF materials are made from the host–guest interaction of ILs, which shares a common dynamic behavior as in liquid electrolytes and MOF as a solid support material. ILs are molten salts composed of charged ions (cations and anions) with a melting point of less than 100 °C, which are non-flammable and have a low volatility, high thermal stability and a tunable design [76]. The cations are usually the derivatives of 1-methylimidazole, but other cations, such as pyridine-based and quaternary ammonium cations, are also typical. Meanwhile, the anions are usually a conjugate base of inorganic acid, such as tetrafluoroborate and hexafluorophosphate. Compared to conventional liquid, the intermolecular forces of ILs are driven by the strong ionic bond that makes ILs have a high lattice energy and melting point. However, ILs made from organic cations have low melting point at room temperature, making it suitable for electric battery application because of its low vapor pressure. A list of common ILs used in battery applications is tabulated in Table 1.

**Figure 2:**
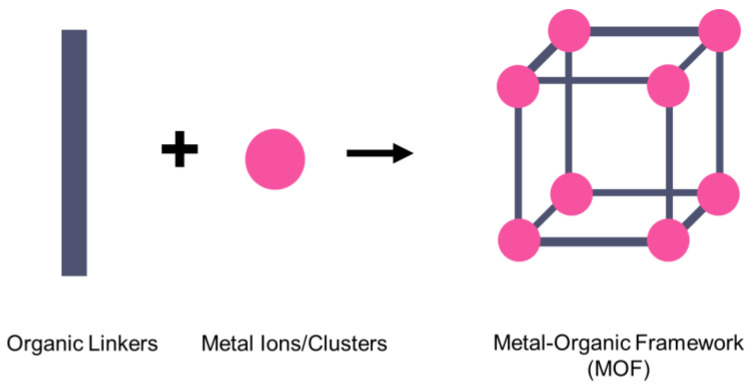
MOF is made of metal ions or metal clusters that are linked to many organic linkers to form a single unit cell of the MOF.

**Figure 3:**
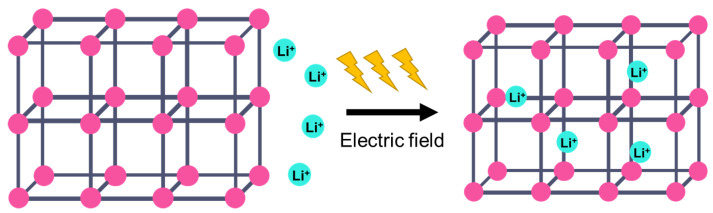
The pore structure of the MOF can provide a transportation channel for the diffusion of ionic species for battery applications.

On the other hand, a metal–organic framework (MOF) is a type of organometallic polymer structure, which is composed of metal clusters linked with organic ligands, as depicted in Figure 2. A MOF is classified as three-dimensional coordination compound because it contains a repeating complex that extends in the x, y and z axes. It can be prepared via self-assembly, which involves the crystallization of metal salt and the organic ligand. The possible intermolecular forces in MOF are van der Waals forces, π–π interactions, hydrogen bonding and stabilization of π-bond by polarized bonds from the synergistic interaction of metal and ligands. The geometry of a MOF is adjustable, depending on the type of metal nodes and length and functional groups of organic ligands. The key features of MOFs are their large pore structure (up to 90% free volume) and big surface area (up to 6000 m^2^/g), which can serve as interaction sites for different ions and molecules. Recently, studies have reported the benefit of confining ILs into the pore of a MOF via host–guest interactions. Because of the thermodynamically unfavorable characteristics of a MOF, it tends to stabilize its 3D structure via the insertion of guest molecules. The guest molecules do not forming a covalent bond with the framework of the MOF, but it undergoes a hydrogen bonding or π-stacking to retain the structure of the composite itself. It is also revealed that loading ILs inside a MOF changes the phase behavior of ILs due to the nanoconfinement effect. The MOF also experiences structural changes in terms of its framework geometry. This IL@MOF hybrid material will open promising applications, especially in carbon dioxide capture, catalysis, gas storage, and especially, solid-state electrolyte for rechargeable batteries (Figure 3).

Shucheng, Zifeng, and Yi reported the success of imidazole incorporation in UiO-67 as a potential superionic conductor candidate [77]. No conductivity was observed for bulk imidazole; however, when incorporating imidazole into MOF, the dynamic motion was accelerated, hence the enhanced ionic conductivity. The recorded ionic conductivity was 1.44 × 10^−3^ S cm^−1^ at 120 °C. The authors highlighted that one of the strategies to increase ionic conductivity is to form a space-charge layer thickness that is equivalent to Debye length (the scale over which mobile charge carriers, such as electrons, screen out electric fields in plasmas and other conductors and how far its electrostatic effect persists) [78,79]. The confinement of imidazole causes hydrogen bond disruption and rearrangement, which provides more pathways for proton migration, thus improving proton conduction. Furthermore, the activation energy decreased as temperature increased due to a low guest–host interaction. The proton conduction was attributed to the Grotthuss mechanism or proton jumping, where excess protons/proton defects diffuse through the hydrogen bond framework of water molecules or other hydrogen-bonded liquids through the formation and accompanying cleavage of covalent bonds involving neighboring molecules [80,81]. However, the structural breakdown of the composite occurs after the leaching test, which suggests that this material might not be stable. Therefore, the further study of the structural and conductivity stability of a MOF@IL is necessary, including testing under different humidity conditions.

**Figure 4:**
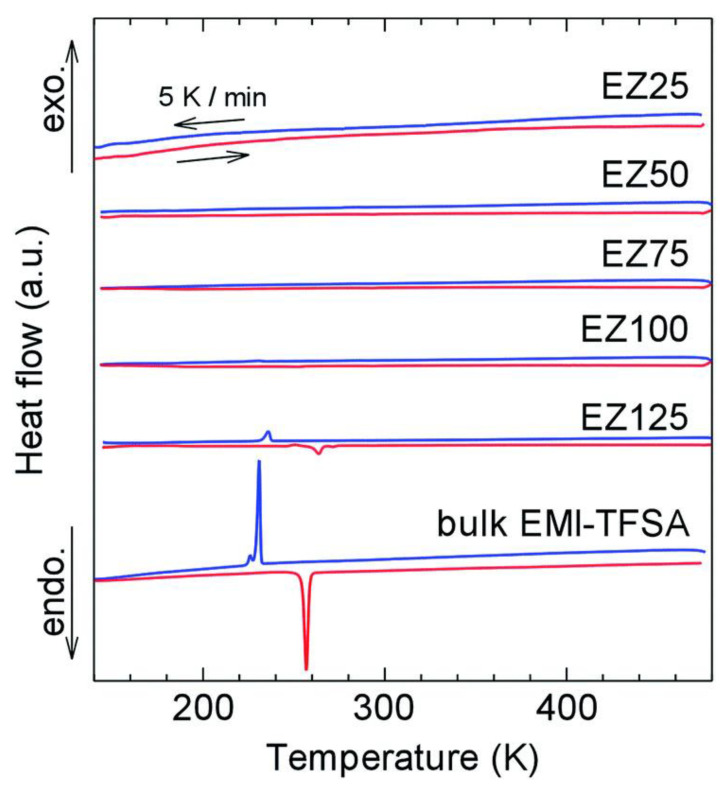
Clear transition phases were observed for bulk EMI-TFSA. The sharp peaks indicate freezing (blue) and melting (red) processes. When confined in ZIF-8, the peaks become smaller and eventually disappear from the thermogram. This was due to the confining effect of the IL inside the pore region of the MOF. Reprinted (adapted) with permission from [82]. Copyright: 2015 The Royal Society of Chemistry.

Meanwhile, Fujie et al. discovered a hybrid EMIM-TFSA@ZIF-8 electrolyte that can conduct electricity at low temperatures [82]. Unlike a pure IL, which tends to solidify when subjected to low temperatures, the nanosized IL inside the MOF was prevented from freezing, as depicted from their DSC analysis in Figure 4. Interestingly, the ionic conductivity was higher in the composite material compared to pristine ZIF-8. The ionic conductivity of the composite was observed below 250 K, which has a comparable value with previously reported liquid electrolytes [83,84,85], making it a suitable electrolyte in LIBs. The maximum entropy method and Rietveld refinement were conducted to visualize the electron density of guest molecules in the porous material and to analyze the structural information of the composite. It was revealed that the charge density contributed by EMIM-TFSA was minimal at the micropore central region, which indicates that the ILs interact strongly with the wall of ZIF-8. Further computational calculation is needed to clearly identify the charge transfer that occurs in the framework. Additionally, the investigation of the performance of this hybrid material in an LIB setup is essential to ensure its compatibility as an alternative SSE.

A report on a lithium-incorporated AMIM-TFSI@MOF-5 as electrolyte in an LIB system was published by Ankit, Raman, and Noriyoshi in 2017 [86]. The synthesized electrolyte was a solidified ion-gel with an ionic conductivity of 1 × 10^−2^ to 2.3 × 10^−3^ S cm^−1^ at 51 °C. The different values of ionic conductivity were due to the different loading percentages of lithium. Furthermore, the cycling performance of the electrolyte in a silicon-based fabricated cell was recorded at 3000 to 3300 mAh g^−1^, which indicates stable-cycling properties with better reversible discharge capacity for a long-term use in an electrical device. On the other hand, the coulombic efficiency was 90%, which speculated the presence of other side reactions during the battery operation. The unwanted reactions may come from the used electrodes while monitoring the electrochemical performance of the LIB. A highly porous electrode with large surface area creates a sufficient electrode–electrolyte interface, leading to a short diffusion pathway [87,88]; however, it also might be the reason of uncontrolled side reactions with electrolytes, leading to degradation in the framework [89]. A deep understanding of the structural properties of the electrode–electrolyte interface will provide insights on the origin of the low coulombic efficiency. Additionally, more studies on the influence of nanoconfined ILs in a MOF are expected in the future to verify whether the presence of these guest molecules deteriorates the efficiency of the generated current.

**Figure 5:**
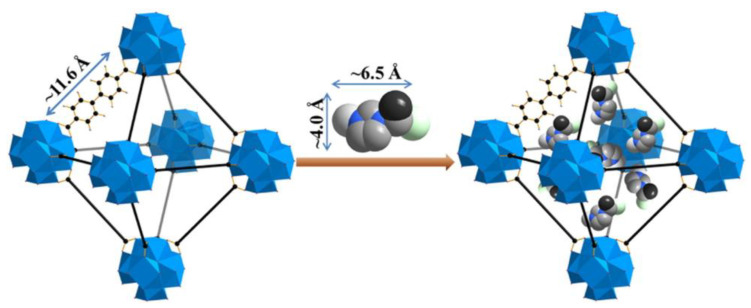
A visualization on the loading of EMIM-Cl IL inside the micropore of UiO-67. The ILs fit perfectly inside the pore structure, allowing ion diffusion for charge transportation. Reprinted (adapted) with permission from [90]. Copyright: 2017 Elsevier.

At the same time, Chen et al. have demonstrated the superior performance of EMIM-Cl@UiO-67 as an electrolyte, which can operate in a high temperature environment [90]. The highest ionic conductivity was 1.67 × 10^−3^ S cm^−1^ at 200 °C with a low activation energy of 0.37 eV. The recorded ionic conductivity was higher compared to a pristine MOF. This might be due to the nanoconfinement effect of ILs inside the micropore of UiO-67. In addition, the dynamic behavior in the composite material differed from the pure IL based on evidence by thermogravimetry analysis and differential scanning calorimetry analysis. The different diffusivity was due to the different phases present at elevated temperatures. The dissimilarity of intermolecular forces in bulk IL and nanoconfined IL might be another factor altering ion mobility. However, further studies on the confinement effect of ILs in nanopores are needed to understand how they flow and how the dynamics of the electrolyte facilitate the transportation of lithium ions. The authors also emphasize the selection of a suitable size of IL as guest molecule as it will influence the ionic conductivity. Figure 5 illustrates the packing of EMIM-Cl inside the UiO-67 support material. Ideally speaking, the IL must be perfectly loaded onto the nanoporous support. If the size of IL is too large, there will be the possibility of blockage inside the micropores of the MOF. On the other hand, if it is too small, the confinement process might be problematic. However, it all depends also on the selection of MOF. With the easy tunability of IL structure and porosity in MOF, the ionic conductivity could be improved tremendously.

**Figure 6:**
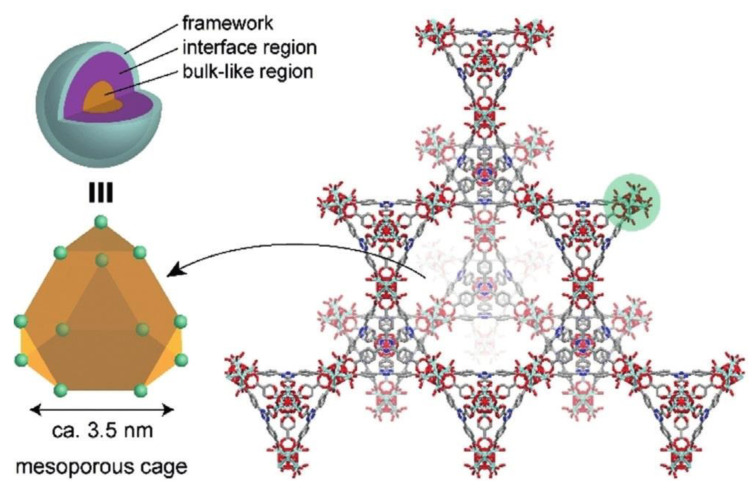
The presence of two different regions in the MOF was due to the large assembly in the MOF structure. The construction of PCN-777 framework led to a hexagonal tunnel that serves as the transportation medium of ions. Reprinted (adapted) with permission from [91]. Copyright: 2019 Wiley Online Library.

In another report, Yoshida et al. synthesized a hybrid EMIM-N(CN)_2_@PCN-777 as a superionic conductor [91]. At room temperature, the recorded ionic conductivity was 4.4 × 10^−3^ S cm^−1^ and the activation energy was 0.20 eV. The ionic conductivity can reach up to 10^−2^ S cm^−1^ at elevated temperatures. Even at a very low temperature, there was no significant loss of ionic conductivity, suggesting its prospective usefulness in operating electrochemical devices at a wide range of temperatures. Due to the large pores of the MOF, there were two distinct regions inside the framework, i.e., the interface region where the interactions of IL and pore surface occurred, and the core region which was fully loaded with bulk IL. Figure 6 clearly illustrated the framework diagram of PCN-777 and both core and interface regions. IL in the core region shows high diffusivity compared to the interface region; therefore, the core-like region in the MOF core is the key to develop a superionic conductor. Moreover, the conductivity of this hybrid electrolyte is better than that of the pristine MOF at both high and low temperatures, eventually surpassing the conductivity of pure ILs.

Additionally, Xu et al. have reported the performance of cyanide-based IL@MOF hybrid electrolytes [92]. Two different cyanide ILs, namely EMIM-SCN and EMIM-DCA, were introduced into MIL-101 to form two different electrolytes. The ionic conductivity recorded for the SCN-type composite was 1.15 × 10^−3^ S cm^−1^ at room temperature and could reach up to 6.21 × 10^−2^ S cm^−1^ at 150 °C. Meanwhile, for the DCA-type composite, the ionic conductivity was 4.14 × 10^−4^ S cm^−1^ at room temperature and increased to 2.45 × 10^−3^ at 150 °C. In general, incorporating SCN as an anionic counterpart could yield a better ionic conductivity compared to DCA. Both anions can fit into the pore of the MOF since their size is smaller than that of the MOF pore. However, the DCA has bigger size compared to SCN, indicating that shorter anions are preferable not only for the perfect fitting in micropores, but also to allow fast ionic transport in the framework. Further studies are recommended to understand the conduction behavior of lithium ions in these two different anions.

Additionally, Pingchun, Mengmeng, and Yanxiang have discovered the promising electrochemical properties of niobium monoxide, an NbO-type MOF incorporated with imidazolium-based IL with different sizes of alkyl chains and anion species [93]. The ionic conductivity values of BMIM-Cl@MOF and EMIM-Br@MOF at 150 °C were 6.63 × 10^−5^ S cm^−1^ and 7.50 × 10^−6^ S cm^−1^, respectively. Based on these finding, a smaller anion, such as Cl, could lead to a higher ionic conductivity than Br. Although the IL loading in Br was higher than Cl and considering the flexible EMIM cation, the size of the anion plays a crucial role in determining the performance of ionic transport in a battery system. A bigger anion might hinder the motion of ions and may cause the framework to disintegrate. Nonetheless, these findings can be further improved by clarifying the effect of cation and anion towards ionic conductivity. For example, ionic conductivity should be tested for EMIM-Cl, EMIM-Br, BMIM-Cl and BMIM-Br to obtain a clearer picture of their generic trend. Moreover, the ionic conductivity of this hybrid material can be further refined by tailoring the porosity of the MOF.

**Figure 7:**
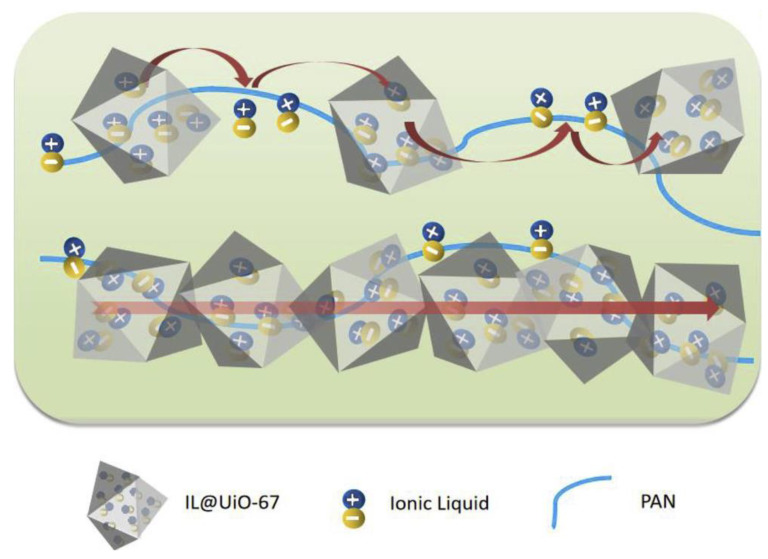
A proposed ion hopping mechanism in the BMIM-BF_4_@UiO-67@PAN membrane electrolyte. The ILs travelled between lattice sites and were facilitated by the PAN matrix. Reprinted (adapted) with permission from [94]. The image was cropped from original source. Copyright: 2020 Elsevier.

Moreover, Chen et al. have reported a novel BMIM-BF_4_@MOF incorporated in a polyacrylonitrile (PAN) as a composite electrolyte [94]. The electrolyte was prepared via the solvothermal synthesis of MOF and followed by a continuously ground with IL. It was then dispersed in DMF together with PAN to form a thin white membrane. The ionic conductivity of 2.53 × 10^−4^ S cm^−1^ at 90 °C was observed with low activation energy (0.6 eV). When the content of ILs were increased from 20% to 60%, the ionic conductivity increased significantly. This indicated that the loading of IL into the MOF has a great influence towards ionic transport. Unconfined IL shows low conductivity because of the strong cation–anion interaction, causing a denser packing of IL and eventually suppressing ion diffusivity. Figure 7 shows the possible ionic transport mechanism in this hybrid composite electrolyte. The authors stated that the ionic conductions mainly occurred inside the micropores of the MOF and along the PAN matrix. Inside the micropores, the activation energy for ionic conduction was quite low compared to the outer region. This was due to the presence of continuous network pathways, which was created from the IL@MOF itself as a nanofiller.

Wu and Go discovered the application of lithium-doped [EMIM][TFSI]@UiO-66 SSE in LIB [95]. The ionic conductivity can reach as high as 3.2 × 10^−4^ S cm^−1^ with a lithium-ion transference number of 0.33 at 25 °C. The interfacial contact between the electrodes and SSE was excellent due to the high surface tension of the nanostructured SSE. The interfacial resistance of Li/SSE and LiFePO_4_/SSE at 60 °C were 44 and 206 Ω cm^2^, respectively. Moreover, the presence of a stable solid electrolyte interphase formed at the Li/SSE interface increases the stability of the lithium plating/stripping process. The electrochemical performance of this SSE was remarkable with high discharge capacities at 130 mAh g^−1^ and a good retention of 100% after 100 cycles at 0.2 C, while the capacities slightly dropped to 119 mAh g^−1^ with 94% retention after 380 cycles at 1 C. These convincing results will open new possibilities for IL@MOF-based SSE for long-lifespan energy storage systems.

Wang et al. studied the electrochemical performance of [EMIM_0.8_Li_0.2_][TFSI]@MOF-525 (Cu) [96]. It showed a superior ionic conductivity of 3.0 × 10^−4^ S cm^−1^ and an enhanced Li^+^ transference number of 0.36 at room temperature. An excellent interfacial compatibility against both electrodes with no significant interfacial resistances was observed. They hypothesized that this was due to the unique interfacial wettability from the nanoconfinement of IL, which provides a 3D Li^+^ conductive network pathway for the movement of conducting ions. When 25 mg cm^−2^ of this SSE loaded to Li/LiFePO_4_ LIB system, the battery performed well with a large temperature window from −20 °C to 150 °C. A capacity of 67 mAh g^−1^ was recorded at 0.05 C, when the LIB system operated at a low temperature (−20 °C), but increased to 145 mAh g^-1^ at 0.5 C when the temperature increased.

Li et al. utilized a Li-[EMIM][TFSI]@HKUST-1 SSE for the development of fast lithium ion transport in LIBs at elevated temperatures [97]. This SSE has a high thermal stability (up to 300 °C) with a relatively good ionic conductivity and Li^+^ transference number of 0.68 × 10^−4^ S cm^−1^ and 0.46 at 25 °C, respectively. The ionic conductivity can increase to 6.85 × 10^−4^ S cm^−1^ when the operating temperature increased to 100 °C. At the same time, the highly stable lithium plating/stripping effectively removed the unwanted reactions, which can prevent the formation of lithium dendrites. The initial discharge capacity was 144 mAh g^−1^ at 0.5 C with a high-capacity retention of 92% after 100 cycles from the assembled LiFePO_4_/SSE LIB system.

Chen et al. reported a novel Li-[Py13^+^][TFSI]@ZIF-67 as SSE in dendrite-free Li metal anodes [98]. The cation of the IL was *N*-propyl-*N*-methylpyrrolidinium (Py13^+^). This electrolyte system has high thermal stability (325 °C) compared to the previous study. Even when exposed to a 1300 °C flame within 60 s, the SSE fails to ignite. The ionic conductivity increased from 0.31 × 10^−3^ S cm^−1^ to 2.29 × 10^−3^ S cm^−1^ when the mass of IL was doubled from 1.0 g to 2.0 g., indicating the vast influence of the concentration of free ions in the IL. The interaction of metal ion of the MOF and TFSI-anions increases the free volume of Li+ ions, which consequently enhances ions mobility. The time-dependent interface stability was measured using the Li/SSE/Li cell at 60 °C. During the first four days, the interfacial resistance decreased and the value remained constant for the next 15 days, suggesting a stable interface of SSE and electrodes without the formation of side reactions. The electrochemical window was 5.4 V vs. Li/Li+, which is higher than organosilicon-group-derived silica-ionogel electrolyte (4.87 V vs. Li^+^/Li) [99] and, when subjected to a wide potential range (−0.5 V to 0.5 V) vs. Li/Li+, the battery was able to resist the oxidation reaction. It was predicted that this type of SSE could solve the lithium dendritic issue, allowing an efficient performance of the LIB in real-life applications.

Table 2 summarizes the list of IL@MOF used as SSE. The values of ionic conductivity and Li+ transference number differ depending on the type of IL@MOF used. The values were also influenced by the operating temperature and the ratio of IL used in the MOF. The IL@MOF with the highest ionic conductivity was [EMIM][SCN]/MIL-101 with an ionic conductivity of 6.21 × 10^−2^ at 150 °C, while the lowest values were those of (1.5[EMIM][Br])/Cu_2_(EBTC)(H_2_O)_2_ and [EMIM_0.8_Li_0.2_^+^][TFSI^−^]/HKUST-1/PEO (7.50 × 10^−6^ at 150 °C and 9.76 × 10^−6^ at 30 °C). The former might be due to the presence of coordinated water ligands, while the latter was due to the absence of IL. Meanwhile, the SSE with the highest lithium ions transference was functionalized UiO-66 (with styrene sulfonate and single Li ion) at 0.9, which is almost near to 1, while the lowest one was [EMIM_0.8_Li_0.2_^+^][TFSI^−^]/HKUST-1/PEO at 0.23 and again it was due to the absence of IL loading. These data show that it is important to incorporate IL inside the micropores of the MOF to provide excellent ionic conductivity and lithium transference number.

## 4. Important Aspects for the Development of the IL@MOF

### 4.1. Stability of the IL@MOF

To ensure that LIBs could perform well, the IL@MOF electrolyte system must possess high thermal stability and be inert to moisture. The framework of the IL@MOF must also not undergo structural transformation to avoid any leaching, which may create problems for the LIB system. Zeeshan et al. reported the thermal stability limit of twenty-nine imidazolium-based ILs combined with ZIF-8 and CuBTC MOF [103]. The main finding from this investigation is that most composites have low thermal stability compared to their pristine MOFs and bulk ILs. They found that the IL@MOF with a functional group in anion sites shows higher thermal stability compared to bulk ILs. Moreover, an increase in alkyl chain length on the imidazolium ring could be the main factor for the decreasing thermal stability. An interesting fact was that the fluorination of the anion site can increase the thermal stability. Among the studied IL@MOF systems, when pairing Cu-BTC with anions of dicyanamide, acetate or phosphate, a significant increase in thermal stability was observed. The stability of Cu-BTC paired with [BMIM][BF_4_] and [BMIM][PF_6_] towards moisture was investigated in another report [104]. The loading of ILs into the framework of Cu-BTC did not offer an observable 3D structure transformation when exposed to water. The thermal stability decreased during the first hour of water exposure; however, when subjected to IL loading, the decomposition of Cu-BTC was slowed down the BET analysis. This shows that, to design a thermally and moisture stable composite, the incorporation of ILs as guest molecules is a must to alter the physical properties of the MOF system.

### 4.2. Optimum IL-to-MOF Ratio

When making the composite of the IL@MOF, one should not ignore the loading ratio of IL to MOF. The optimum amount of IL loading ensures that the selective pores of the MOF are occupied and enhance the ionic conductivity. The correct mass loading of IL also governs the solid-state properties of the IL@MOF due to the nanoconfinement of liquid IL. A recent study by Zhang et al. revealed that the ionic conductivity of [BMIM][TFSI] in HKUST-1 MOF was influenced by the loading of ILs [105]. The ionic conductivity was increased when the percentage of [BMIM][TFSI] was elevated from 15% to 80%. The value was even higher when using a pelletized form (which referred to nanowetted interfaces) for the ionic conductivity measurement. As the percentage of IL increased from 80% towards 100%, the ionic conductivity dropped. The phenomenon was previously described in a molecular simulation that was related to the IL bunching inside the micropore of HKUST-1. This result shows that the ability of the IL@MOF to conduct ions could achieve the best performance even if the pores of the MOF are not fully occupied. In practical applications, only a little amount of ILs is needed to power electronic devices.

### 4.3. The Safety of IL@MOF Materials

Although many studies revealed the promising performance of the IL@MOF as SSE, the safety of the IL@MOF material is rarely discussed. The absence of an organic solvent in the IL@MOF reduces the risk of explosion in LIBs; however, if using a less stable MOF, the leaching of metal ions could cause another problem. Meanwhile, the toxicity of the MOF is also seldomly explored. Table 3 and Table 4 summarize the hazard properties of the selected MOF and IL. While the toxicity in MOF is less profound, the usage of IL might be problematic as some of it is quite toxic. A group of experts from the *Institut Catala de Nanociencia i Nanotecnologia*, Barcelona (Spain), conducted in vitro and in vivo toxicity assessment of a series of MOFs [106]. They concluded that the most toxic MOF was HKUST, followed by ZIF-8, MIL-101, MIL-100 and ZIF-7. The least toxic MOFs were Mg-MOF-74, Co-MOF-74, UiO-66 and UiO-67. The degradation of MOD in the cell culture solution releases the leaching metal ions that firmly verified the toxicity of MOF. Additionally, other factors that can cause the toxicity level of MOF were the formation of other species during degradation and the different crystal parameters, such as the size, shape and charge. The influence of the molecular and extended solid-state system of the MOF was, however. unclear and we anticipate that future investigation will be carried out to understand the toxicity of MOFs. On the other hand, most ILs are toxic, but designing a safer IL could minimize the problem. For example, Romero et al. investigated imidazolium-based ILs with two alkyl substituents at the R1 and R2 positions (R1: fixed methyl group, R2: the different length of alkyl chain) and a counter ion with different anions (Cl^−^, PF_6_, XSO^4−^) [107]. All ILs were not biodegradable in the considered conditions, but the toxic level was low when the length of alkyl chain at R2 was short. The anions also did not directly influence the toxicity of ILs. In the case of the IL@MOF, we believe that no experts have studied it to date.

## 5. Computational Modeling and Its Application in Battery Research

Most of the research in material chemistry involves phenomena that occur at a microscopic level. Relying solely on an experimental approach might not produce the desired properties. By using computational modelling methods, the structure and properties of the materials can be accessed and calculated [108,109,110,111,112]. The two main approaches in computational modelling are the quantum mechanical approach and classical mechanical approach. Quantum mechanical simulations of materials are based upon the Schrödinger equation that describes the probability of finding an electron in a quantum mechanical system. The method does not depend on the fitting parameters because all structural information is calculated by solving the Schrödinger equation. This approach is also known as ab initio methods, which means “from first principles” or “from the beginning” [113,114]. However, in certain conditions, a semi-empirical approach is used as an alternative cost-effective tool in the simulation of large molecules. Meanwhile, the classical mechanical employs empirical parameters, and usually involves force field to describe the molecular properties and dynamics of materials [115]. Compared to the quantum mechanical approach, which treats electrons of each atom in the calculation, in classical mechanics, each atom is simulated as a single particle. Whichever method is used, the main objective of computational modelling is to simulate the atomistic behavior of materials by using correct algorithms and approximations [116,117,118].

### 5.1. Density Functional Theory (DFT)

One of the quantum mechanical methods to describe the electronic properties of materials in many-body systems, condensed phases and molecules is density functional theory (DFT). It is based on an ab initio calculation where the prediction of the electronic structure is conducted by solving the Schrödinger equation. The simplest form of the Schrödinger equation is indicated in (1):

ĤΨ = EΨ(1)

The three main components in this equation are the Hamiltonian operator (Ĥ), energy eigenvalue (E) and wave function (Ψ). This equation is also called time-independent Schrödinger equation. Ĥ operator on the left expresses the Hamiltonian acting on Ψ(x). The Hamiltonian operator describes the kinetic energy (T), potential energy from external field due to Coulomb repulsion of the two nuclei (V) and coulombic interaction between electrons (U), such as in (2):

(T + V + U)Ψ = EΨ(2)

This equation is approximated via the Born–Oppenheimer approximation (adiabatic principle), which assumes that the dynamics of atomic nuclei and electrons in a molecular system can be treated separately due to the fact that the mass of nuclei is heavier than that of electrons [119,120,121]. By *freezing* the nuclear positions, the complex variables in the Schrödinger equation can be reduced from 4 (N_e_ + N_n_) to only 4 N_e_, which eventually simplify the Hamiltonian term, making it easier to compute the desired wavefunction and structural properties, especially in supramolecules. However, the U term complicates the many-particle equation by disallowing simplification into a single-particle equation.

Before DFT was popularized, the simplest Hartree–Fock method was introduced to solve the many-electron wavefunction via Slater determinants [122,123,124]. The method utilized the Hartree potential, but also forces exchange interactions by forcing the antisymmetricity of the electronic wavefunction. This is to minimize the total binding energy of the atoms by making sure that electrons of parallel spin stay away from each other. The problem with this assumption is that it ignores correlations in the dynamics between two electrons with anti-parallel spins without the full incorporation of the electronic correlation, resulting in higher energy in a system.

In DFT, the many-electron wavefunction is solved by computing the electron density. The functional in DFT is the electron density, which is a function of space and time. It was described by two Hohenberg–Kohn (H-K) theorems in 1964. The first H–K theorem stated that the ground state density uniquely determines the external potential of a system because the external potential is a unique functional of electron density in the ground state, therefore the total energy is also a functional of the ground state electron density [125,126,127]. Because of this theorem, all properties of a system are calculated only from the ground state electron density. In the second H–K theorem, the total energy of a system is minimized from the correct ground state energy.

The method of computing ground-state density of a system was formulated by Kohn and Sham. The aim of the Kohn–Sham method is to calculate the ground state density as a real system by replacing a fully interacting system to a non-interacting one [128,129]. The exact ground state density of a N-electron system is calculated from the individual electron wavefunction (3):

ρ(r) = 2 ∑ φ_i_∗φ_i_ (r) (3)

where single-particle wavefunctions, ψ, are the N lowest-energy solutions of the Kohn–Sham equations. The fictitious Kohn–Sham system has all functionals of the charge density, such as in (4):

[T + V(r) + V_H_ (r) + V_XC_ (r)] φ_i_ (r) = ε_i_ φ_i_ (r) (4)

where V(r) represents the external potential, V_H_(r) is the classical electrostatic Hartree term (the functional is rewritten as (5)), and V_XC_(r) is the exchange-correlation energy (6), which includes the non-classical electrostatic interaction energy and the difference between the kinetic energies of interacting and non-interacting systems. Some of the commonly used approximation methods include local density approximation (LDA), generalized gradient approximation (GGA), and adiabatic connection method (ACM). The selection of a suitable V_XC_(r) functional for a simulated system is crucial because it determines the accuracy of the DFT calculation.

V_H_ (r) = e^2^ {(∫ ρ (r′))/|r-r′|} d^3^ r′ (5)

V_XC_ (r) = (∂E_XC_ [n])/∂n(r) (6)

**Figure 8:**
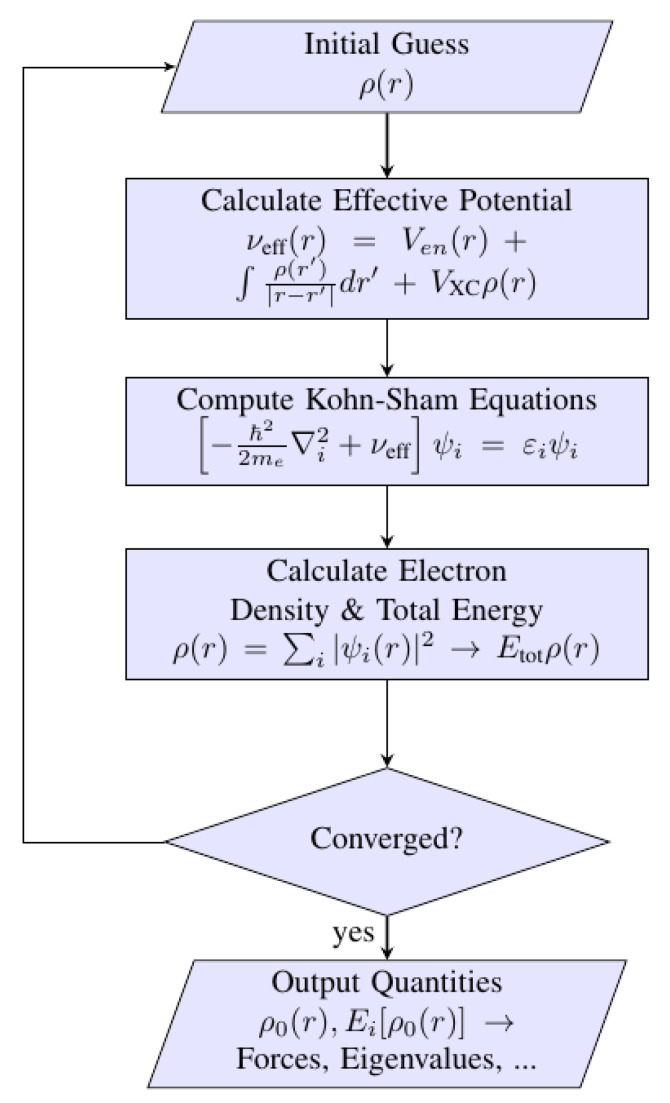
Algorithm to calculate the Kohn–Sham equation. First, electron density is guessed as an input to calculate the effective potential, ν_eff_ (r), which is made by Coulombic interactions of electron and nuclei with an exchange-correlation functional. Next, the diagonalization of the Kohn–Sham equation is made before producing the electronic density together with total energy of the system. The iterative calculation is continued until the criterion of convergence is achieved by including the last ρ (r) rather than the initial guess. The desired electronic properties are printed after the criterion is satisfied.

The Kohn–Sham equation is calculated via the iterative method using a self-consistent loop, such as in Figure 8. The constructed algorithm minimizes the energy configuration corresponding to the atomic coordinates by searching the stationary points of a function whose exact form is mainly unknown. The quasi-Newton method and conjugate gradient method are examples of iterative calculation used to search for the minimum energy of a system [130,131,132].

### 5.2. Molecular Dynamics Simulation

In addition to DFT, a molecular system can be simulated via molecular mechanics through a classical approach. The most common approach is molecular dynamics (MD) simulation, which calculates the movements of atoms in a molecular assembly via Newton’s second law of motion [133,134]. In classical MD, all particles are treated explicitly, such as atom and ion, and are typically assigned with van der Waals radius, constant net charge and polarizability. Bonded particles are simulated as *springs* and the value of equilibrium distances are calculated from the experimental bond length. Examples of information that can be extracted from MD simulations include dynamical properties of system, transport coefficients, time-dependent responses to perturbations, spectrum and rheological properties. 

In MD simulations, the Born–Oppenheimer approximation is assumed valid and, since nuclei are treated as classical particles, they experience electrons as an average field. The motions of molecules are based on Newton’s equation of motion (7):

F_i_ = m_i_ a_i_ (7)

where F_i_ is the force acted on the particle i, m_i_ is the mass particle i and a_i_ is its acceleration. Forces also related to the negative derivative of potential energy, such as in (8). Solving forces in three-dimensions returns into the gradient of the potential as depicted in (9).

F_i_ = −dU/dx = −∇_i_ U (8)

−(dU/dx) x − (dU/dy) y − (dU/dz) z(9)

Therefore, the equation of forces and potential energy can be expressed in (10):

−dV/dr = m(d^2^r)/(dt^2^) (10)

where the negative derivative of potential energy is equal to the changes in position as a function of time.

Equations of motion need to be solved to interpret the dynamics that occur in a simulated system. It is unfeasible to solve it analytically because the motions of all particles are coupled; therefore, the practical method to use is finite difference, which approximates derivatives with finite differences. The positions and velocities of particles are approximated as a Taylor series, and Verlet algorithm is used to construct a sequence of points r that closely approach the points r(t) on the trajectory of the exact solution [135,136]. In this case, the positions are approximated to the second derivative in (11) and (12):

r(t + Δt) = r(t) + Δtν(t) + 1/2 Δt^2^ a(t) (11)

r(t − Δt) = r(t) − Δtν(t) + 1/2 Δt^2^ a(t) (12)

In this method, the velocity term does not appear as it was eliminated after addition process. The estimation of the velocity is needed so that the kinetic energy of the simulated system can be calculated. Another variation of the Verlet method is the Leap-Frog Verlet method, which is time reversible and could reflect the physical reality of certain simulation problems. In this algorithm, the position, velocity and acceleration are computed simultaneously because of the incorporation of the velocity, which solved the first time step equation in the Verlet algorithm. The second step is to derive a(t + ∆t) from the interaction potential. The final step is to calculate the velocity (13):

ν(t + Δt) = ν(t) + 1/2 (a(t) + a(t + Δt))Δt(13)

The conditions of a simulated system, such as temperature and pressure, need to be specified because different phenomena require a different set of ensembles. Since the equation of motion is related to potential energy, one could say that the total energy (E) of the system is conserved (14):

E = E_kinetic_ + E_potential_ (14)

The common ensemble for MD simulation is a microcanonical ensemble, which contains a fixed number of particles (N), constant volume (V) and constant energy [137,138,139]. The ensemble is usually denoted as NVE. For a system with a constant pressure or temperature environment, NVE is the suitable ensemble. Another type is the canonical ensemble (NVT), where the number of particles, volume and temperature (T) are kept constant. Meanwhile, an isothermal–isobaric ensemble (NPT) fixes the particles, pressure (P) and temperature in a system. An appropriate ensemble is desirable to ensure all thermodynamic properties are well defined.

The next aspect of MD simulations is force field. Force field is a set of equations and an accompanying constant that are used to estimate the forces, specifically the potential energy between atoms in a molecule (intramolecular forces) and also between molecules (intermolecular forces). In general, the total potential energy is a combination of bonded terms and non-bonded terms (15). For bonded terms (16), it consists of bond energy, angle energy, dihedral angle and improper angle, while non-bonded terms (17) include electrostatic interactions and Van der Waals interaction. There are also interactions that result from non-bonded interactions, such as hydrogen bonds and a hydrophobic effect.

U_total_ = U_bonded_ + U_non-bonded_ (15)

U_bonded_ = U_bond_ + U_angle_ + U_dihedral_ + U_improper_ (16)

U_non-bonded_ = U_electrostatic_ + U_van der Waals_ (17)

To conclude, the basic steps in MD simulations are:

1. Create an initial state of particles;
2. Introduce interaction potentials;
3. Predict how the particles move.

**Figure 9:**
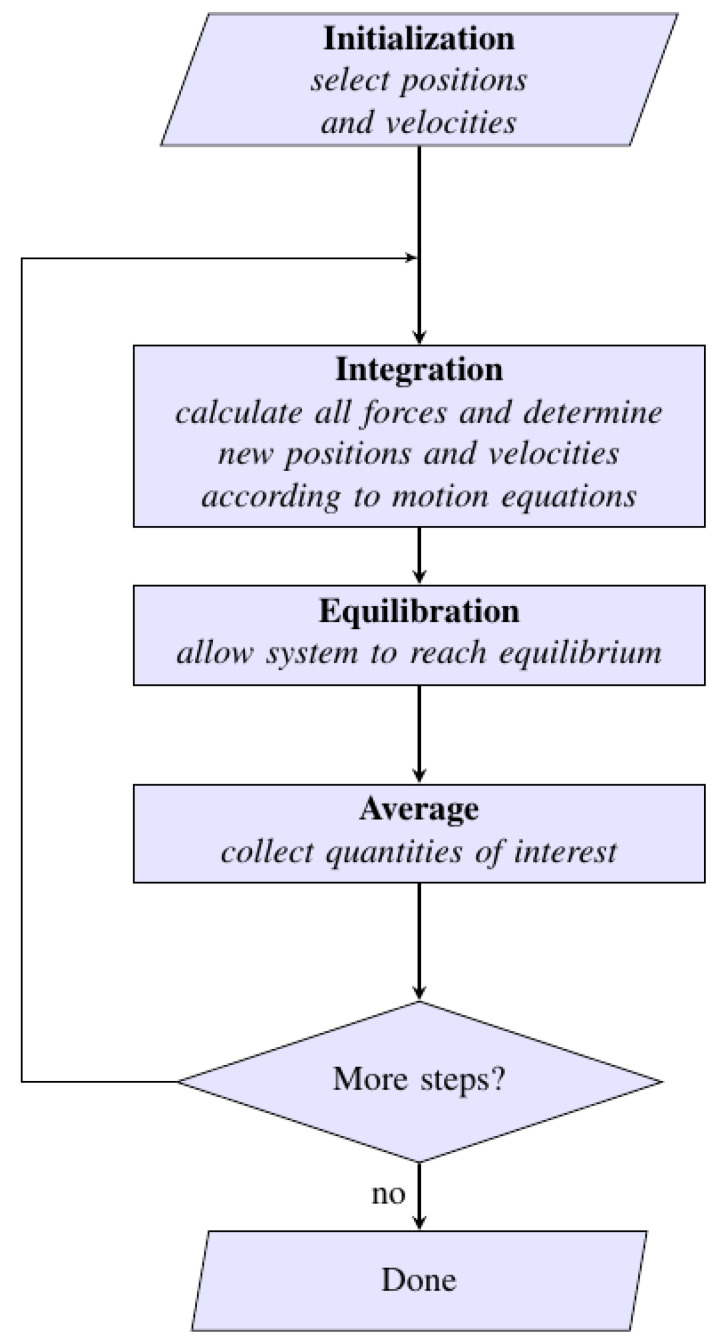
A simplified algorithm of MD simulations. The main goal is to simulate dynamics in a molecular system as a function of time after an energy input is incorporated at equilibrium. In the first step, after specifying the conditions of the run (N, T, V and time step), the positions and velocities of all atoms in the system are initialized. After that, all forces are computed from the interaction potential. The system continues to run until it reaches equilibration, and finally the thermodynamic averages, positions and velocities are analyzed.

An algorithm of MD simulations step is illustrated in Figure 9.

### 5.3. Recent Computational Studies of the IL@MOF as a Promising Electrolyte

**Figure 10:**
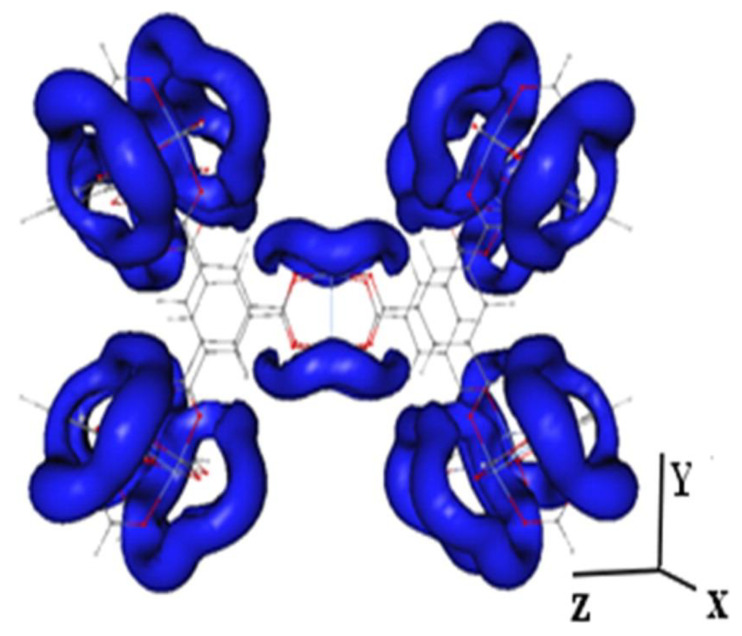
Isosurface of MESP in the Cu-BTC structure at V = −52.51 kJ mol^−1^. The blue region indicates the distribution of electron density.

Some researchers have applied DFT calculation and MD simulations to study the structural properties of IL@MOF material. Dhumal et al. applied the DFT method to investigate the molecular interactions of a copper-based MOF with imidazolium-based ionic liquid [140]. A three-step analysis was performed: the investigation of possible interaction sites and the influence of IL loading towards structural change in MOF; the investigation of the effect of MOF towards a IL pair; and the examination of the integrated picture of the simulated IL@MOF. As shown in Figure 10, the isosurface of the molecular electrostatic potential (MESP) was plotted to identify the interaction sites of IL within the vicinity of MOF micropores. A symmetrical distribution of electron density was observed inside the MOF structure where the electrons were mainly delocalized around oxygen atoms of the joint molecules. Two possible molecular interactions within the MOF framework were hydrogen from cationic site of IL to oxygen atom from linkers, and oxygen to MOF or oxygen to cation of IL. The authors revealed that the interaction of the IL and MOF originated from the charge-transfer phenomenon or redistribution of electron density based on the DFT calculation [141,142]. They also observed that, when the IL content was increased, the repulsive forces within the IL also increased.

**Figure 11:**
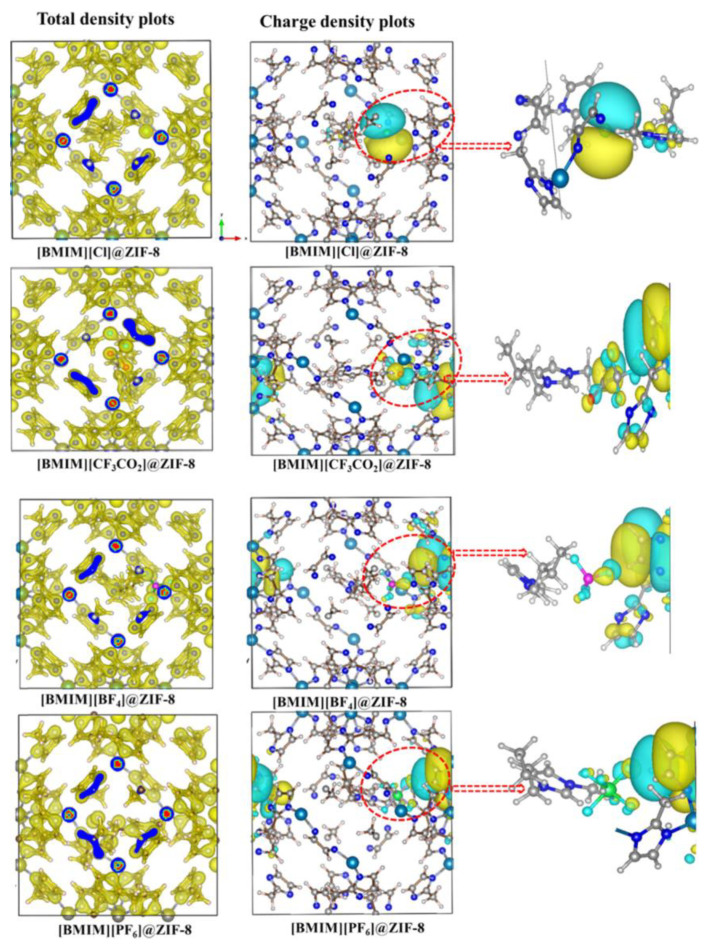
Total electron density and charge density of different ZIF-8 with different anions (Cl, CF_3_CO_2_, BF_4_ and PF_6_) were seen on the isosurface plots. The yellow region denotes the charge depletion, while blue region indicates accumulation of charge. Reprinted with permission from [143].

Meanwhile, Thomas et al. reported the effect of hydrophilicity and hydrophobicity of anion counterparts in ILs towards the stability of ZIF-8 [143]. The cation of ILs used was BMIM^+^, while the studied anions were Cl^−^, CF_3_CO_2_^−^, BF4^−^ and PF6^−^. The author stated that the nature of bonding and dispersive interaction at the interface are the two key factors that lead to a stable IL@MOF. The interpretations of this computational calculation provide important insights into the design of hybrid materials with many applications, including catalytic conversion at confinement, gas storage and separation techniques. The authors varied the conditions of the DFT computation first before calculating the structural properties of the IL@MOF. An illustration of the charge transfer of different anions with ZIF-8 can be observed in Figure 11. The Cl^−^ anion shows a weak interaction towards the MOF based on the blue shifts observed in the IR spectra, which corresponds to a lesser charge transfer. For other anions that comprise the fluorine atom, the charge transfer was large, which can be seen from the red shift. This might be correlated to high electronegativity in fluorine compared to chlorine. The highest magnitude of the charge transfer belongs to CF_3_CO_2_^−^, which might be due to the presence of the electron withdrawing group in carboxylate.

From the electron density analysis, only weak bonded intermolecular forces (dispersive and van der Waals) were present in the IL@MOF. Compared to Cl^−^ where most of the electron density accumulated in IL, the fluorinated IL@MOF shows the localization of charge density on the surface of the MOF and low dispersion in anion species. The interaction between imidazole carbon (C_2_) and the anionic part of ZIF-8 indicates a stable charge transfer, thus confirming the suitability of imidazolium-based IL with ZIF-8. The authors concluded that the tendency of IL to interact with MOF is due to the similar behavior, i.e., the hydrophobicity of both components.

**Figure 12:**
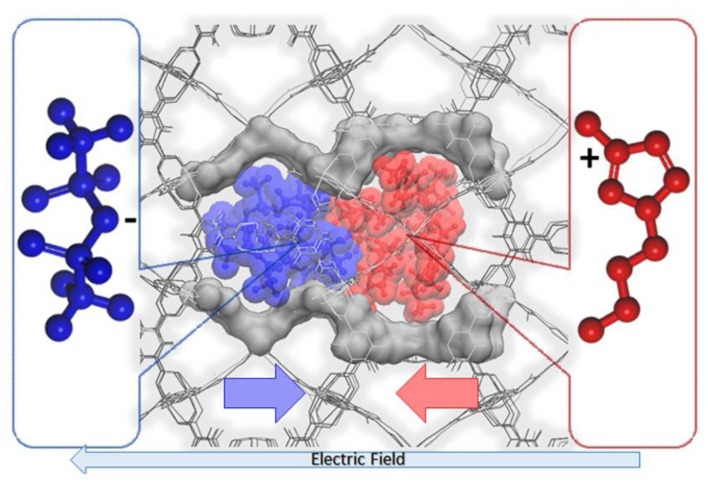
An illustration of the blocking in the MOF pore caused by both cation (blue) and anion (red). Reprinted (adapted) with permission from [100]. Copyright: 2019 American Chemical Society.

In another report, Kanj et al. highlighted the need to investigate the ionic conduction behavior in the IL@MOF [100]. The dynamic aspects are one of the important properties, especially in the charging–discharging process of supercapacitors and batteries. Here, a hybrid IL@MOF material made from [BMIM][NTf_2_] as IL and HKUST-1 as MOF was simulated via MD simulations using a UFF4MOF force field, which was designed specifically for the MOF simulation [144]. The main finding here is the decrease in ionic mobility, which is correlated to the drift velocities of the oppositely moving cations and anions. When 20% of IL was loaded in the pore of MOF, the ionic conductivity started to decrease. This was verified by the MD simulations. With the minimum loading of ILa, there is a large free volume, and the anions and cations were moving in opposite directions with negligible interaction. However, as ILs were increased, they occupied the space in the pores and were forced to collide with each other, blocking the ionic transport. The blockage of the micropore of the MOF was induced by the electric field, as shown in Figure 12. This phenomenon was clearly observed at high IL loading where a non-uniform overall density of ion appeared on high- and low-density areas. This is called ‘bunching’, where an initially homogenous distribution of IL becomes inhomogeneous.

**Figure 13:**
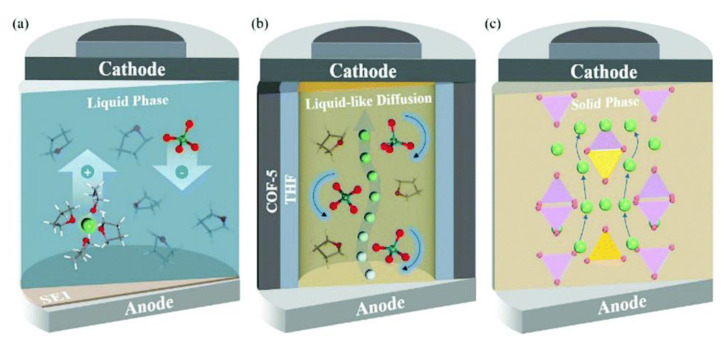
Representative diagram of Li^+^ ion conduction in (**a**) liquid LIB, (**b**) COF LIB and (**c**) ISE LIB. In a conventional liquid LIB, the movement of Li^+^ ions is facilitated by the mixture of solvent, while in ISE, Li^+^ undergoes the ion hopping mechanism. The COF possess both mechanisms with liquid-like diffusion. Reprinted (adapted) with permission from [145]. Copyright: 2019 The Royal Society of Chemistry.

Since there is limited literature on Li^+^ ion diffusivity in IL@MOFs, in this review we included one report that utilized a carbon-organic framework (COF). Zhang et al. investigated the computational details of 2D COF in mixtures of LiClO_4_ as source of Li^+^ ions and THF as solvent for lithium-ion battery applications [145]. The ab initio MD method was used, which incorporates the first principle calculation together with empirical data to simulate the dynamics of the material. The COF exhibits a 2D structure placed in a stack manner, which led to the formation of tunnels with nanoconfined ClO_4_ and THF. The AIMD calculation reveals several essential findings for the design of a fast-conducting solid electrolyte. The main finding is that the ion conduction mechanism of Li^+^ inside this COF is considered as liquid, even though the COF is regarded as a solid material. Instead of the ion hopping mechanism, which is common in other inorganic solid electrolytes, the movement of Li^+^ is facilitated by the simultaneous interaction of Li^+^ with both ClO_4_^−^ and THF. The ionic conductivity value was extracted from the Nernst–Einstein equation with a value of 0.30 mS cm^−1^ at 300 K, which is similar to the experimental value (0.26 mS cm^−1^). From the probability density analysis, the distribution of ClO_4_^−^ mainly resides in the center of the tunnel (which was also verified by solid-state NMR [146]), which occupies a radius slightly smaller than the Li ions, which in turn means a strong interaction exist between them. On the other hand, the distributions of THF in the tunnel are in the center and near the wall of the COF. The region where THF is near to the wall can act as a lubricant for the wettability of the Li^+^ ions and the COF, and prevent the hold back effect of the solid walls on Li^+^ ion diffusion and ClO_4_^−^ rotation. A representative diagram of Li^+^ ion diffusivity in three different LIBs is illustrated in Figure 13. We can say that the ionic conduction mechanism of Li^+^ ions in this material is a hybrid mechanism of ionic diffusion in liquid electrolytes and solid inorganic electrolytes. However, sluggishness in ionic conductivity may be a challenging problem probably due to the improper design of the COF electrolyte, especially inside the micropore framework where Li^+^ ion diffusion mainly occurs inside this region. Some future strategies can be implemented, such as varying the amount of solvent, the use of different Li salts and the use of different radii of tunnel of COF materials. Reducing the size of anion and enlarging the pore size of COF ensure excellent Li ion conduction.

## 6. Future Directions

The Coronavirus disease (COVID-19) has changed the way we live since 2020. The global pandemic has affected almost all of our daily tasks. The extensive demand of electronic devices, such as laptops and tablets, demands the urgent transition to reliable, high-energy density and safe batteries. Moreover, the demand for medical devices has increased in the treatment of COVID-19 patients. The rapid growth of miniaturized electronic devices with flexible, thin and large surface areas has pushed academicians and industries to search for promising and reliable materials for the development of energy storage. Such developments have already been achieved in the development of ultrathin and uniform T-Nb_2_O_5_ microsphere anodes for high performance LIB systems [147]. SSEs are now slowly being implemented in LIBs because they are highly stable, non-flammable and could offer outstanding performance. IL@MOF systems show promise as it can be manufactured as SSEs, additives, and electrode materials for future applications [148]. The rapid demand of safe and powerful energy will escalate the manufacturability and scalability of solid-state batteries within a few years. According to Huang et al. [149], the scalability of solid-state batteries is influenced by the consequences of material selection, including the availability, scaling capacity and fluctuating price of materials, the process to transform these materials to fabricate batteries, and the electrochemical performance that may be expected with these selected materials. The choice of IL@MOF will influence the overall flow process and upscaling of solid-state battery, and to ensure the applicability of this type of SSE, industrial simulation process pilot studies are expected, from the bulk production of IL@MOFs to the manufacturing of marketable LIBs. Last, but not least, more studies are needed to investigate the recyclability of LIBs. A battery contains a number of heavy metals and toxic chemicals and, without proper disposal, soil contamination and water pollution will become problematic. Some valuable materials in batteries can be recycled; however, the recycling of electrolytes is quite challenging as most of the electrolytes need to undergo through several separation processes in order to be able to recover the original electrolytes. The recycling of IL@MOFs might be possible since it is in solid-state form compared to the conventional liquid electrolytes.

## 7. Conclusions

The need of new SSEs in LIBs is necessary to overcome the drawbacks of liquid electrolytes. IL@MOFs are proposed as suitable SSEs due to their tunable functionality and electrochemical properties. Based on the current literature, most of the researchers employ imidazolium-based IL as the guest molecule to be incorporated inside the micropores of MOFs. The commonly used MOFs as host materials are UiO-67, ZIF-8 and MOF-5. The ionic conductivity of IL@MOFs is better than that of bulk ILs or pristine MOFs, which is associated with the nanoconfinement of ILs in MOFs. The main factors that govern the excellent ionic transport are the size of the pore in MOFs, the IL loading and the size of anion. The commonly proposed ionic mechanism is the ion hopping mechanism, which is similar to SPEs. From a computational perspective, some insights can be gained from the structural properties analysis and dynamic simulations. Some MOFs show blockage inside the pore due to the unfitted size of anions, which in reality may inhibit ionic transport. Moreover, different anions have distinctive charge transfer inside the MOF, which influence the chemical stability of the IL@MOF. Understanding the atomistic level of certain properties, such as electronic band structure, density of states, how ILs are nanoconfined in a support material and the dynamics of lithium-ion diffusion in IL@MOFs, is still difficult due to the limited computational studies of IL@MOFs in the literature. Additionally, there is the need to further explore the essential electrochemical properties of IL@MOFs assembled in a prototype LIB cell, which includes, but is not limited to, the working potential window, transference number, electrochemical window and the resistance towards dendrite growth.

## Figures and Tables

**Table 1:** Common ILs used for the formation of IL@MOFs in battery electrolyte applications.

Ionic Liquid	Name	Abbreviation
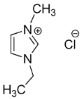	1-ethyl-3-methylimidazolium chloride	EMIM-Cl
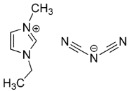	1-ethyl-3-methylimidazolium dicyanamide	EMIM-DCA
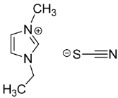	1-ethyl-3-methylimidazolium thiocyanate	EMIM-SCN
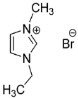	1-ethyl-3-methylimidazolium bromide	EMIM-Br
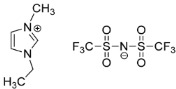	1-ethyl-3-methylimidazolium bis(trifluoromethylsulfonyl)imide	EMIM-TFSI
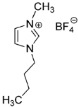	1-butyl-3-methylimidazolium tetrafluoroborate	BMIM-BF_4_
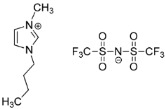	1-butyl-3-methylimidazolium bis(trifluoromethylsulfonyl)imide	BMIM-TFSI
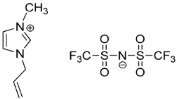	1-allyl-3-methylimidazolium bis(trifluoromethylsulfonyl)imide	AMIM-TFSI

**Table 2:** Summary of the IL@MOF used in the literature review.

IL@MOF	Ionic Conductivity (S/cm)	Li+ Transference Number	Ref.
HKUST-1/[BMIM][TFSA]	45 ± 20 nS m^2^ mol^−1^ (20% IL pore loading and less)	-	[100]
UiO-67(Zr)/[EMIM][Cl]	1.67 × 10^−3^ at 200 °C	-	[90]
[BMIM][BF4]/UiO-67(Zr)/PAN	2.53 × 10^−4^ at 90 °C	-	[94]
Imidazole/UiO-67	1.44 × 10^−3^ at 120 °C	-	[77]
[EMIM][TFSA]/ZIF-8	-	-	[82]
[EMIM][N(CN)_2_]/PCN-777	4.4 × 10^−3^ at 298 K >10^−2^ above 343 K	-	[91]
[EMIM][SCN]/MIL-101	6.21 × 10^−2^ at 150 °C 1.15 × 10^−3^ at 25 °C	-	[92]
[EMIM][DCA]/MIL-101	2.45 × 10^−3^ at 150 °C 4.14 × 10^−4^ at 25 °C	-	[92]
(1.5[BMIM][Cl])/Cu_2_(EBTC)(H_2_O)_2_	6.63 × 10^−5^ at 150 °C	-	[93]
(1.5[EMIM][Br])/Cu_2_(EBTC)(H_2_O)_2_	7.50 × 10^−6^ at 150 °C	-	[93]
Li-incorporated [AMIM][TFSI]/MOF-5	1 × 10^−2^ to 2.3 × 10^−3^ at 51 °C (Values varied due to different Li% loading)	-	[86]
[EMIM_0.8_Li_0.2_^+^][TFSI^−^]/HKUST-1/PEO	1.20 × 10^−4^ at 30 °C 9.76 × 10^−6^ at 30 °C (without IL)	0.36 (with IL) 0.23 (without IL)	[101]
Functionalized UiO-66 (with styrene sulfonate and single Li ion)	6.0 × 10^−5^ but improved to 7.8 × 10^−4^ after adding ethylene carbonate + propylene carbonate (25 °C), 7.9 × 10^−5^ (60 °C), 1.1 × 10^−4^ (90 °C)	0.9	[102]
HKUST-1@[EMIM][TFSI]-Li	0.68 × 10^−4^ (25 °C) 6.85 × 10^−4^ (100 °C)	0.46 (25 °C) 0.68 (100 °C)	[97]
[EMIM][TFSI]@UiO-66	3.2 × 10^−4^ S cm^−1^	0.33	[95]
[EMIM_0.8_Li_0.2_][TFSI]@MOF-525 (Cu)	3.0 × 10^−4^ S cm^−1^	0.36	[96]
Li-[Py13^+^][TFSI]@ZIF-67	2.29 × 10^−3^ S cm^−1^	-	[98]

**Table 3:** The danger properties of MOF. - = low; O = medium; + = high. The data were retrieved from the Material Safety Data Sheet (MSDS).

MOF	Flammability	Toxic	Corrosive	Harmful	Irritant	Oxidizing
HKUST-1 (Cu-BTC)	-	O	-	+	+	-
UiO-66	-	-	-	O	+	-
UiO-67	-	-	-	O	+	-
ZIF-8	-	O	+	+	-	-
MIL-101	-	-	-	-	+	-

**Table 4:** The danger properties of IL. - = low; O = medium; + = high. The data were retrieved from the Material Safety Data Sheet (MSDS).

IL	Flammability	Toxic	Corrosive	Harmful	Irritant	Oxidizing
[EMIM][Cl]	O	O	-	+	+	-
[EMIM][DCA]	-	O	+	+	+	-
[EMIM][SCN]	O	+	O	+	O	-
[EMIM][Br]	-	O	+	O	+	-
[EMIM][TFSI]	-	O	+	O	+	-
[BMIM][BF_4_]	O	+	+	+	+	-
[BMIM][TFSI]	-	+	-	+	+	-
[AMIM][TFSI]	-	-	-	O	+	O
